# Genetic association and causal effects between inflammatory bowel disease and conjunctivitis

**DOI:** 10.3389/fimmu.2024.1409146

**Published:** 2024-09-04

**Authors:** Shuangqing Chang, Qinghua Luo, Zhifang Huang

**Affiliations:** ^1^ Department of Anorectal Surgery, Jiangmen Wuyi Hospital of Traditional Chinese Medicine, Jiangmen, China; ^2^ Clinical Medical College, Jiangxi University of Chinese Medicine, Nanchang, China

**Keywords:** inflammatory bowel disease, conjunctivitis, genetic association, genetic risk loci, genetic structure

## Abstract

**Background:**

Inflammatory bowel disease (IBD) is often clinically associated with conjunctivitis, which may result from genetic associations and causal effects.

**Methods:**

Genetic correlations were investigated through the genome-wide association study (GWAS) data on IBD and conjunctivitis using the linkage disequilibrium score regression (LDSC) and heritability estimated in summary statistics (HESS). The causal effect analysis was performed using four methods of Mendelian randomization (MR) and the genetic risk loci common to both diseases were identified by the statistical method of conditional/conjoint false discovery rate (cond/conjFDR), followed by genetic overlap analysis. Finally, a multi-trait GWAS analysis (MTAG) was performed to validate the identified shared loci.

**Results:**

IBD (including CD and UC) and conjunctivitis showed a significant overall correlation at the genomic level; however, the local correlation of IBD and CD with conjunctivitis was significant and limited to chromosome 11. MR analysis suggested a significant positive and non-significant negative correlation between IBD (including CD and UC) and conjunctivitis. The conjFDR analysis confirmed the genetic overlap between the two diseases. Additionally, MTAG was employed to identify and validate multiple genetic risk loci.

**Conclusion:**

The present study provides evidence of genetic structure and causal effects for the co-morbidity between IBD (both CD and UC) and conjunctivitis, expanding the epidemiologic understanding of the two diseases.

## 1 Introduction

Inflammatory bowel disease (IBD) is a recurrent immune-mediated disease with two subtypes, ulcerative colitis (UC) and Crohn’s disease (CD), both characterized by chronic diarrhea, abdominal pain, and hematochezia (1) with certain differences in their lesion patterns. Specifically, UC occurs mostly in the rectosigmoid colon showing fine granulomatous changes in the mucosa, whereas, CD mainly involves the right colon and ileum with cobblestone-like changes (2). According to the reports, 6.8 million people worldwide affected with IBD were reported in 2017 with a loss of 1.02 million healthy life years due to the resulting disability, thereby ranking 4th among digestive diseases (3). In addition, understanding the extra-intestinal manifestations (EIM) of IBD is crucial, such as lesions in the eyes, joints, liver, and mucous membranes of the skin (4). Reportedly, the prevalence of ocular complications in patients with IBD is 3.5–11.8%. Of these, conjunctivitis is the most common ocular condition characterized by inflamed and swollen conjunctival tissues with vascular congestion, ocular discharge, and pain (5, 6). Presently, the co-morbidity of IBD and conjunctivitis is one of the major public health concerns. Therefore, analysis of the genetic perspective of their common genetic risk loci may be effective in disease management.

Although the investigation of the causal relationship between IBD and conjunctivitis has been facilitated by the improvement of genome-wide association studies (GWAS) in recent years (7, 8). Based on GWAS data, a two-sample Mendelian randomization (MR) analysis has demonstrated that IBD elevates the genetic predisposition to allergic conjunctivitis (9). Furthermore, there is sporadic evidence indicating that certain pleiotropic loci may contribute to a potential association between these conditions. For instance, the *NOD2* gene, which heightens susceptibility to IBD, may also play a role in the pathogenesis of vernal keratoconjunctivitis through its overexpression (10). Nevertheless, the investigation into shared genomic loci between IBD and conjunctivitis remains sparse and underexplored. While analyzing the genetic structures shared by two or more diseases, the intersection of their respective positive GWAS significant loci often generates negative results due to the phenomenon of polygeny (11). Besides, increasing the sample size to obtain significant results will require more personnel, money, and time (12). Recently, several novel and reliable genetic statistical methods have emerged that allow effective analysis of the genetic correlations between the traits of two diseases. These methods have been successfully employed in the study of the correlations between glaucoma and depression (13), schizophrenia and cognitive level (14), and IBD and psoriasis (15). Therefore, a similar approach was utilized in the present study to improve the understanding of the genetic association between IBD and conjunctivitis.

Briefly, IBD and conjunctivitis were initially analyzed to determine their genetic correlations overall and locally using the linkage disequilibrium score regression (LDSC) (16) and heritability estimated in summary statistics (HESS) (17), respectively. Since MR, also known as a natural randomized controlled trial (RCT), is based on the “random assignment of parental alleles to offspring”, it eliminates the possible confounding factors. Therefore, MR was used for causality analysis in the present study (18). Finally, the identification of genetic risk loci between traits and the analysis of genetic correlations of multiple overlapping genes were performed using the conditional/conjoint false discovery rate method (con/conjFDR) (19). We also conducted a multi-trait genome-wide association study (MTAG) to validate the identified shared loci (20).

## 2 Materials and methods

### 2.1 GWAS data

Three datasets, including IBD (ID: ebi-a-GCST004131, *N*_case_=25,042, *N*_control_=34,915), CD (ID: ebi-a-GCST004132, *N*_case_=12,194, *N*_control_=28,072), and UC (ID: ebi-a-GCST004133, *N*_case_=12,366, *N*_control_=33,609) from the IEU GWAS database (https://gwas.mrcieu.ac.uk/) were selected based on the sample size, number of SNPs, study ethnicity (Europe), and year of publication. Moreover, GWAS data for conjunctivitis (*N*_case_=32,417, *N*_control_=28,895) were obtained from the FinnGen database (https://r10.finngen.fi/) (21).

### 2.2 Genetic correlation analysis

LDSC (version 1.0.1) measures the degree of genetic effects shared by two traits and estimates their genetic correlation (or r_g_) (22). It is the most significant parameter in the LDSC analysis and reflects the degree of association and effect. The first step of the LDSC analysis converted the GWAS summary statistics of the traits into the LDSC representation according to the relevant and default parameters of munge_sumstats.py. The second step calculated genetic correlations for the traits using the −r_g_, -ref-ld-chr, and -w-ld-chr parameters as reference. The tool (https://alkesgroup.broadinstitute.org/LDSCORE/) provided pre-calculated linkage disequilibrium (LD) score files for the -ref-ld-chr and -w-ld-chr markers. In addition, the LD reference panel used for the analysis was derived from the European pedigree information from the 1000 Genomes Project (23).

### 2.3 Local genetic correlation analysis

HESS calculates local SNP heritability and measures the degree of similarity between two traits driven by genetic variation (17). The process of calculating localized genetic correlations was as follows. First, the genes encoding the trait under study were divided into 1,703 segments that were not correlated with a pre-specified LD, followed by a chromosome-by-chromosome data processing. Finally, the correlation analysis was performed at the chromosomal level corresponding to the two traits. In addition, the statistically significant correlation of the HESS results was defined as p < 0.05/1703 = 2.94E−05 corrected with the Bonferroni method.

### 2.4 Mendelian randomization analysis

Potential causal associations between IBD and conjunctivitis were evaluated using a bidirectional two-sample MR analysis following its three main assumptions (24). In addition, the causal effects were estimated mainly by the inverse variance weighted (IVW) (25), while MR-Egger (26), weighted median (WM) (27), and maximum likelihood (ML) (28) methods supplemented the findings. In addition, pleiotropic (26, 29), heterogeneity (30), and leave-one-out (31) analyses were performed to ensure the quality, accuracy, and reliability of the analyzed results. The entire MR analysis utilized the TwoSampleMR R (https://mrcieu.github.io/TwoSampleMR/) and the MR-PRESSO R software packages (https://github.com/rondolab/MR-).

### 2.5 Conditional quantile–quantile plots

The conditional quantile-quantile (QQ) plot depicted the cross-phenotypic polygenic enrichment for two different traits. When the proportion of SNPs associated with a primary phenotype (e.g., IBD) continually increased with the strength of association with a secondary phenotype (e.g., conjunctivitis), an enrichment relationship was present between the two (32). Each QQ plot demonstrated the distribution of the P value for the primary phenotype, which was determined by its correlation with the secondary phenotypes and was categorized as P < 0.10, P < 0.01, and P < 0.001. The QQ plots were plotted using Python 3.5 at the precimed package (https://github.com/precimed).

### 2.6 CondFDR/ConjFDR analysis

The condFDR/conjFDR method for gene identification determined specific common loci not exceeding the significance threshold based on GWAS data for both traits (33). The condFDR methods were statistically based on the Bayesian method, which generally identifies loci associated with major phenotypes according to the association of secondary phenotypes (34). Initially, the test statistics were reordered and subsequently, associations between these variants and the primary phenotypes were recalculated. Further, the primary and secondary phenotypes were subjected to a backward process to obtain the backward condFDR values for both traits. Next, the condFDR analysis was performed to identify the common genetic loci.

To ensure a more accurate FDR related to the two traits, the highest value of condFDR was selected as the conjFDR value. Eventually, gene loci associated with the two phenotypes were detected. The conjFDR was analyzed following the standard procedure mentioned on the website (https://github.com/precimed/pleiofdr).

### 2.7 Functional analysis

The condFDR/conjFDR analysis generated novel, common, and specific loci. For subsequent positional mapping and functional annotation, the functional mapping and annotation (FUMA) protocol (https://fuma.ctglab.nl/) was used. The screening criteria for independent significant SNPs were set to “conjFDR < 0.05” and “r2 < 0.06”. In addition, the approximate LD of r2 < 0.1 indicated lead SNPs. These mapped genes were subjected to functional enrichment analysis using the enrichment analysis tool of the Sangerbox platform (http://vip.sangerbox.com/) (35).

### 2.8 Cross-trait meta-analysis

We performed an MTAG analysis between IBD and conjunctivitis using Python 3.11.5. Compared to conventional single-trait GWAS analysis, MTAG leverages multi-trait GWAS summary statistics for joint analysis, enhancing statistical power. Furthermore, compared to other multi-trait whole-genome analysis methods, it has a wider range of applicability (20).

## 3 Results

### 3.1 Genetic correlation

The results of LDSC analysis of IBD and conjunctivitis suggested a genetic correlation Z-score of 4.58 and a rg value of 0.22 (p = 4.59e−06 < 0.05). Similarly, the Z-score and rg of CD were 4.28 and 0.22 (p = 1.84e−05 < 0.05), respectively. The rg for UC was smaller (0.16, p = 4.1e−03 < 0.05) than that of IBD and CD. Consequently, a positive association between either IBD or subtypes of IBD and conjunctivitis was observed.

**Figure 1:**
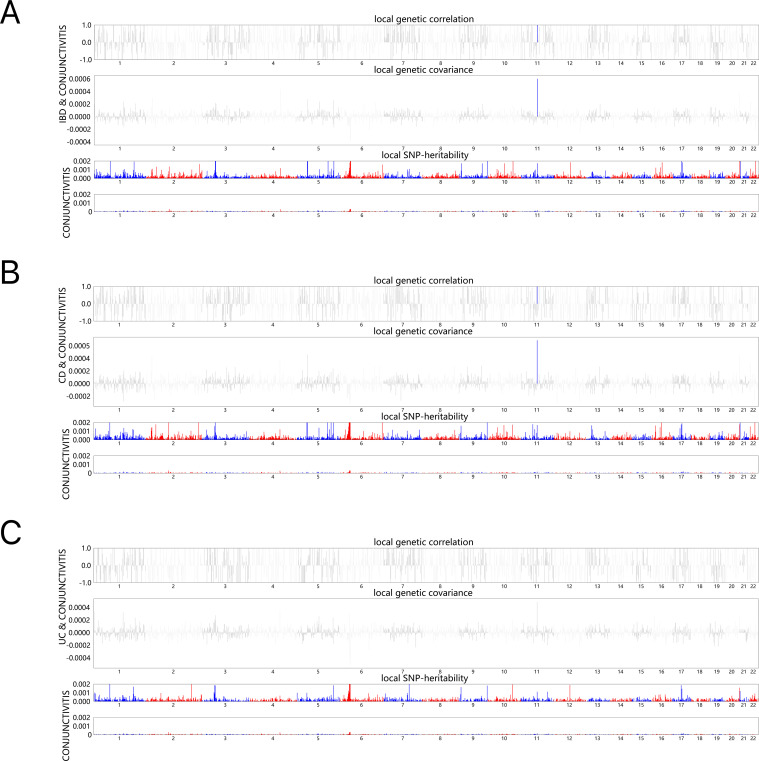
HESS analysis of conjunctivitis and IBD, CD and UC. The top and middle sections of each subgraph represent local genetic correlations and covariances, respectively, and the colored bars represent loci with significant local genetic correlations and covariances. The bottom portion represents the local snp heritability of an individual trait, and the colored bars represent loci with significant local snp heritability. **(A)** Local genetic correlation between IBD and conjunctivitis. **(B)** Local genetic correlation between CD and conjunctivitis. **(C)** Local genetic correlation between UC and conjunctivitis. IBD, inflammatory bowel disease; CD, Crohn’s disease; UC, ulcerative colitis.

Next, the local genetic correlation map showed a genetic overlap of IBD and CD with conjunctivitis on chromosome 11; however, the results for UC were not significant (**Figures 1A–C**). The resultant data for specific analyses are shown in **Supplementary Tables S1–S3**.

### 3.2 Mendelian randomization

**Tables 1**, **2** demonstrate the final results of the correlation analysis between IBD (including CD and UC) and conjunctivitis using four methods. The forward MR analysis (IBD and subtypes as exposure and conjunctivitis as outcome) suggested a positive causality in all three cases (p < 0.05, **Figures 2A–C**, **Table 1**). Conversely, the backward MR analysis did not demonstrate a causal effect of conjunctivitis on IBD or its subtypes (**Table 2**). Moreover, none of the six MR analyses showed significant pleiotropy (**Tables 1**, **2**). The F-statistics corresponding to all the instrumental variables were > 10, indicating no bias in the weak instrumental variables. Further, in the leave-one-out analysis (**Figures 3A-F**), SNPs showed a concentrated distribution without evidence of any abnormal SNPs. The details of the instrumental variables used in this study are mentioned in **Supplementary Tables S4–S9**.

**Table 1:** MR analysis of the causal association between IBD (including CD and UC) and conjunctivitis.

Exposures	Outcomes	nSNPs	Method	OR (95%CI)	P	Heterogeneity test			Pleiotropy test	F
						Method	Q	P	P intercept	
IBD	Conjunctivitis	90	IVW (mre)	1.05 (1.03-1.08)	7.49E-06	MR Egger	171.73	2.73E-07	0.55	29.86-500.60
			WM	1.06 (1.02-1.09)	3.68E-04	IVW	172.41	2.82E-07		
			MR Egger	1.04 (0.97-1.10)	0.25					
			ML	1.06 (1.04-1.07)	3.7E-02					
CD	Conjunctivitis	73	IVW (mre)	1.04 (1.02-1.06)	5.37E-04	MR Egger	161.90	4.70E-09	0.80	30.15-489.58
			WM	1.02 (1.00-1.05)	0.06	IVW	162.04	6.91E-09		
			MR Egger	1.03 (0.97-1.09)	0.29					
			ML	1.04 (1.02-1.05)	2.23E-07					
UC	Conjunctivitis	47	IVW (mre)	1.03 (1.00-1.06)	0.03	MR Egger	97.43	9.83E-06	0.74	30.47-186.78
			WM	1.04 (1.00-1.07)	0.02	IVW	97.67	1.38E-05		
			MR Egger	1.02 (0.92-1.11)	0.04					
			ML	1.03 (1.01-1.05)	9.49E-03					

IBD, inflammatory bowel disease; CD, Crohn’s disease; UC, ulcerative colitis. IVW (mre), Inverse variance weighted (multiplicative random effects); IVW (fe), Inverse variance weighted (fixed effects); WM, Weighted median; ML, Maximum likelihood.

**Table 2:** MR analysis of the causal association between conjunctivitis and IBD (including CD and UC).

Exposures	Outcomes	nSNPs	Method	OR (95%CI)	P	Heterogeneity test			Pleiotropy test	F
						Method	Q	P	P intercept	
Conjunctivitis	IBD	6	IVW (fe)	1.07 (0.87-1.31)	0.51	MR Egger	4.00	0.41	0.75	31.23-50.61
			WM	1.17 (0.92-1.49)	0.19	IVW	4.12	0.53		
			MR Egger	0.89 (0.29-2.67)	0.84					
			ML	1.07 (0.88-1.31)	0.51					
Conjunctivitis	CD	5	IVW (fe)	1.31 (1.00-1.72)	0.70	MR Egger	3.42	0.33	0.38	32.25-50.61
			WM	1.37 (0.97-1.92)	0.34	IVW	4.63	0.33		
			MR Egger	3.16 (0.58-17.27)	0.35					
			ML	1.32 (1.00-1.73)	0.70					
Conjunctivitis	UC	6	IVW (fe)	1.01 (0.78-1.31)	0.81	MR Egger	2.53	0.64	0.74	31.23-50.61
			WM	1.06 (0.78-1.44)	0.83	IVW	2.66	0.75		
			MR Egger	1.30 (0.32-5.30)	0.78					
			ML	1.01 (0.78-1.31)	0.69					

IBD, inflammatory bowel disease; CD, Crohn’s disease; UC, ulcerative colitis. IVW (mre), Inverse variance weighted (multiplicative random effects); IVW (fe), Inverse variance weighted (fixed effects); WM, Weighted median; ML, Maximum likelihood.

**Figure 2:**
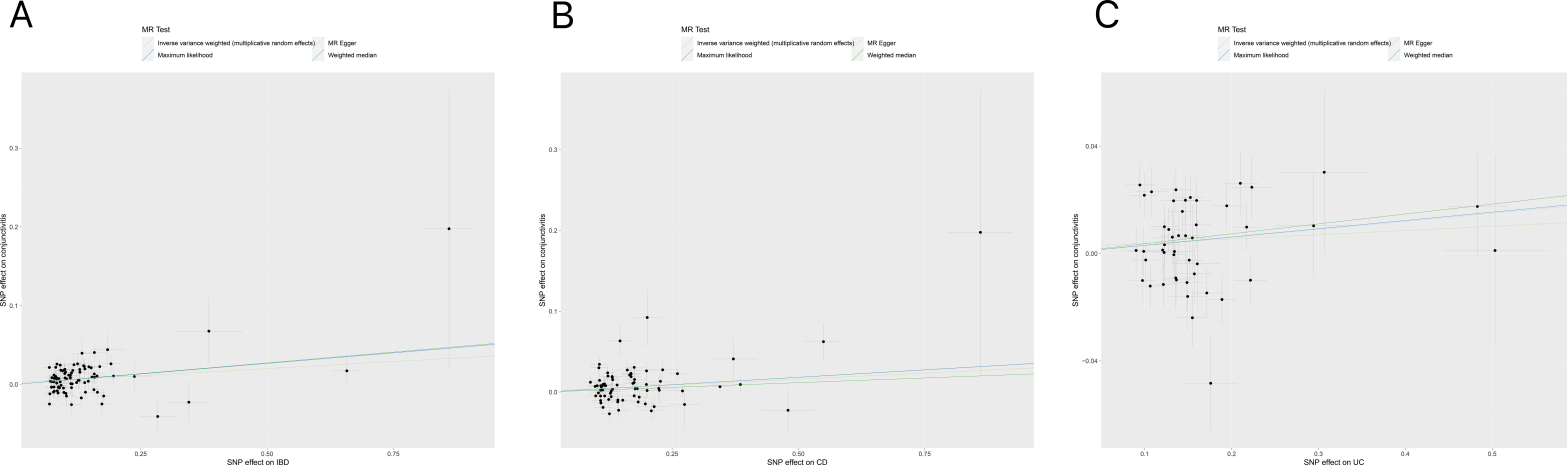
**(A)** Scatter plot for MR analyses of the causal effect of IBD on conjunctivitis. The slope of each line corresponding to the estimated MR effect per method. **(B)** Scatter plot for MR analyses of the causal effect of CD on conjunctivitis. **(C)** Scatter plot for MR analyses of the causal effect of UC on conjunctivitis. IBD, inflammatory bowel disease; CD, Crohn’s disease; UC, ulcerative colitis.

**Figure 3:**
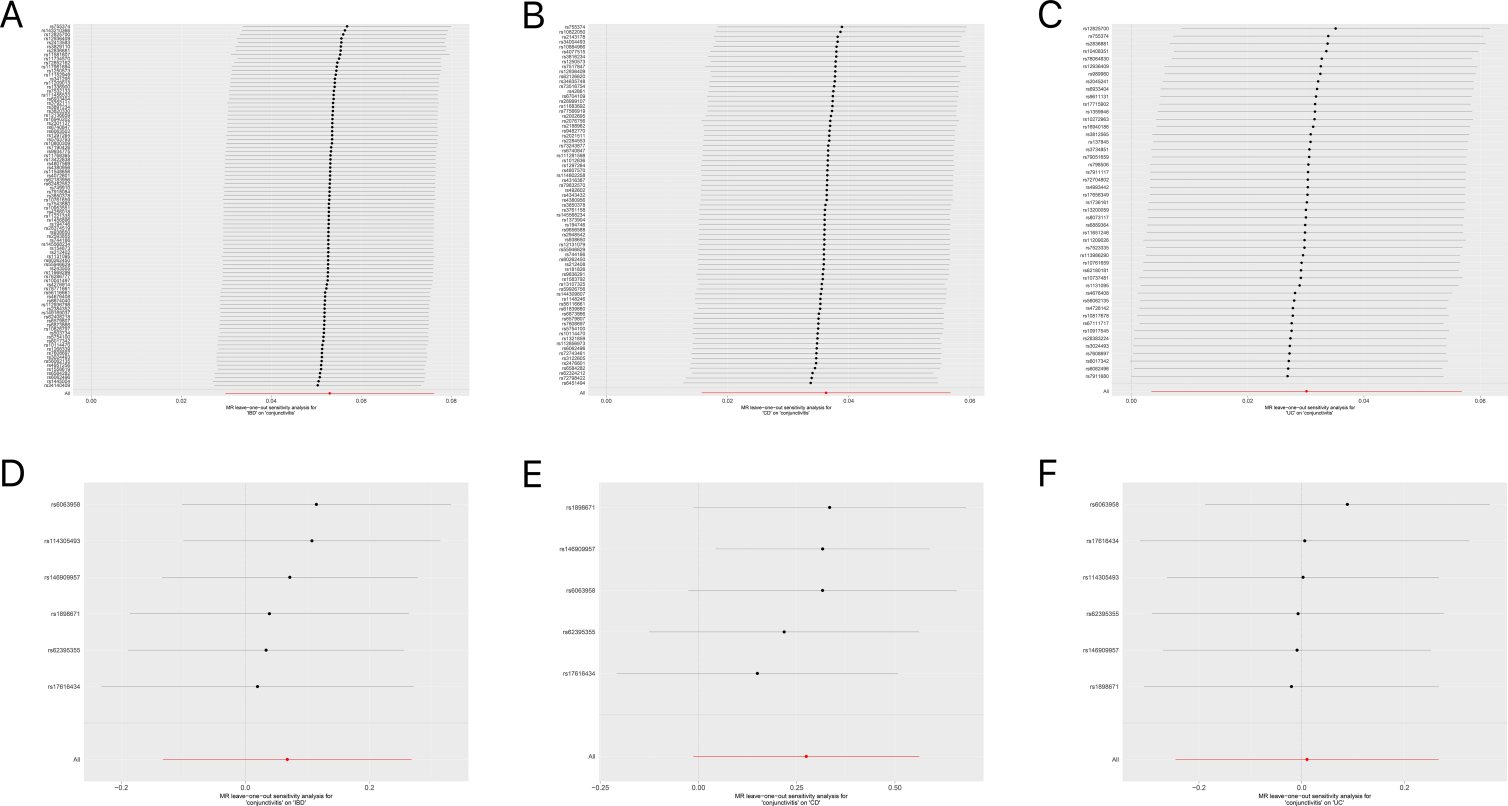
**(A)** Forest plot for the leave-one-out analysis of IBD on conjunctivitis. **(B)** Forest plot for the leave-one-out analysis of CD on conjunctivitis. **(C)** Forest plot for the leave-one-out analysis of UC on conjunctivitis. **(D)** Forest plot for the leave-one-out analysis of conjunctivitis on IBD. **(E)** Forest plot for the leave-one-out analysis of conjunctivitis on CD. **(F)** Forest plot for the leave-one-out analysis of conjunctivitis on UC. IBD, inflammatory bowel disease; CD, Crohn’s disease; UC, ulcerative colitis.

### 3.3 Common genomic loci between IBD (including CD and UC) and conjunctivitis identified by ConjFDR analysis

**Figure 4:**
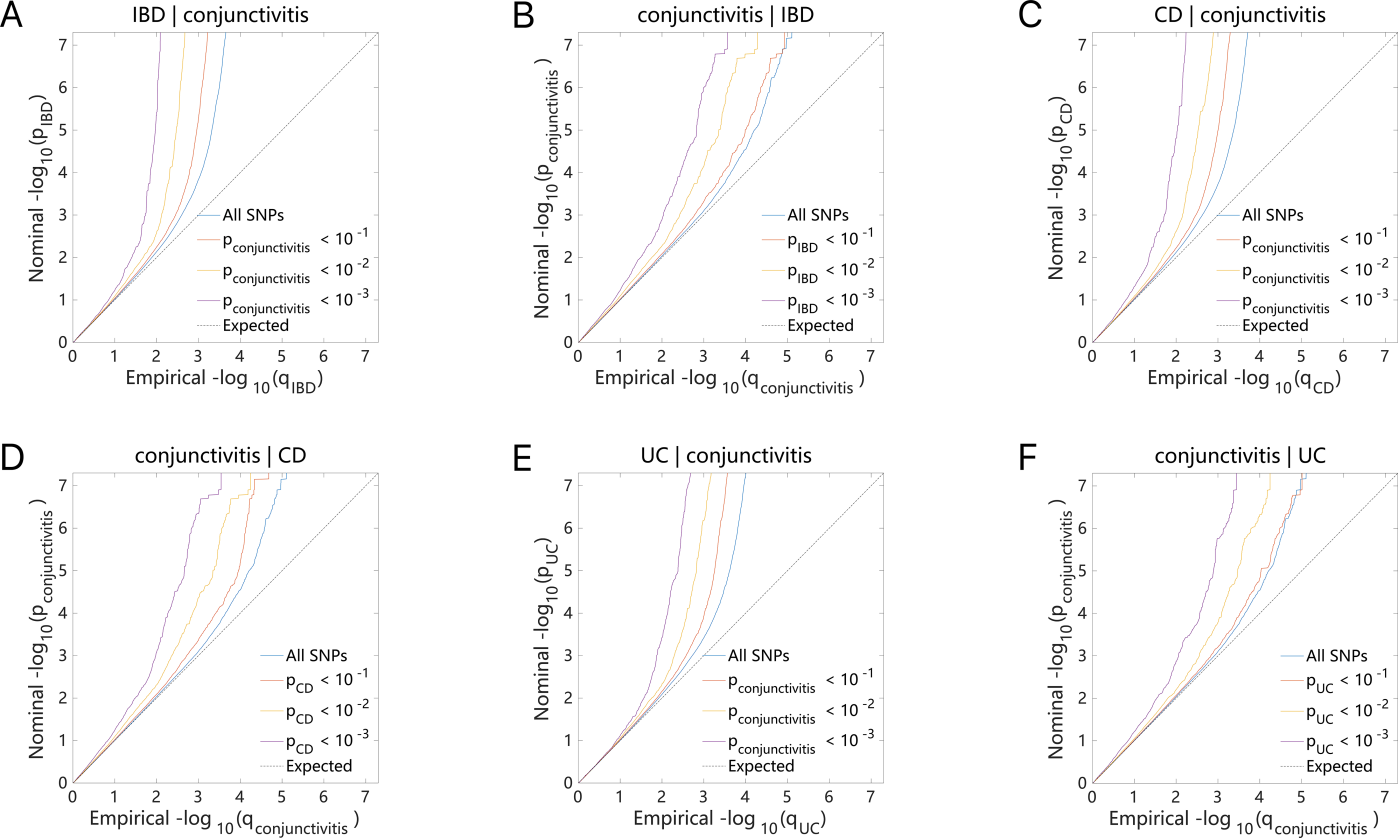
Conditional quantile-quantile plot. The dashed line indicates the expected line under the null hypothesis, and the deflection to the left indicates the degree of pleiotropic enrichment. **(A)** IBD-conjunctivitis. **(B)** conjunctivitis-IBD. **(C)** CD-conjunctivitis. **(D)** conjunctivitis-CD. **(E)** UC-conjunctivitis. **(F)** conjunctivitis-UC. IBD, inflammatory bowel disease; CD, Crohn’s disease; UC, ulcerative colitis.

The Q-Q plot (**Figures 4A–F**) indicated that either IBD (including CD and UC) or conjunctivitis constantly shifted to the left as the association p-value decreased. This phenomenon suggested a strong correlation between the IBD subtypes (including CD and UC) and conjunctivitis, in which multiple genetic loci overlap. Thus, they exhibited genetic enrichment while sharing the same genetic structure.

**Figure 5:**
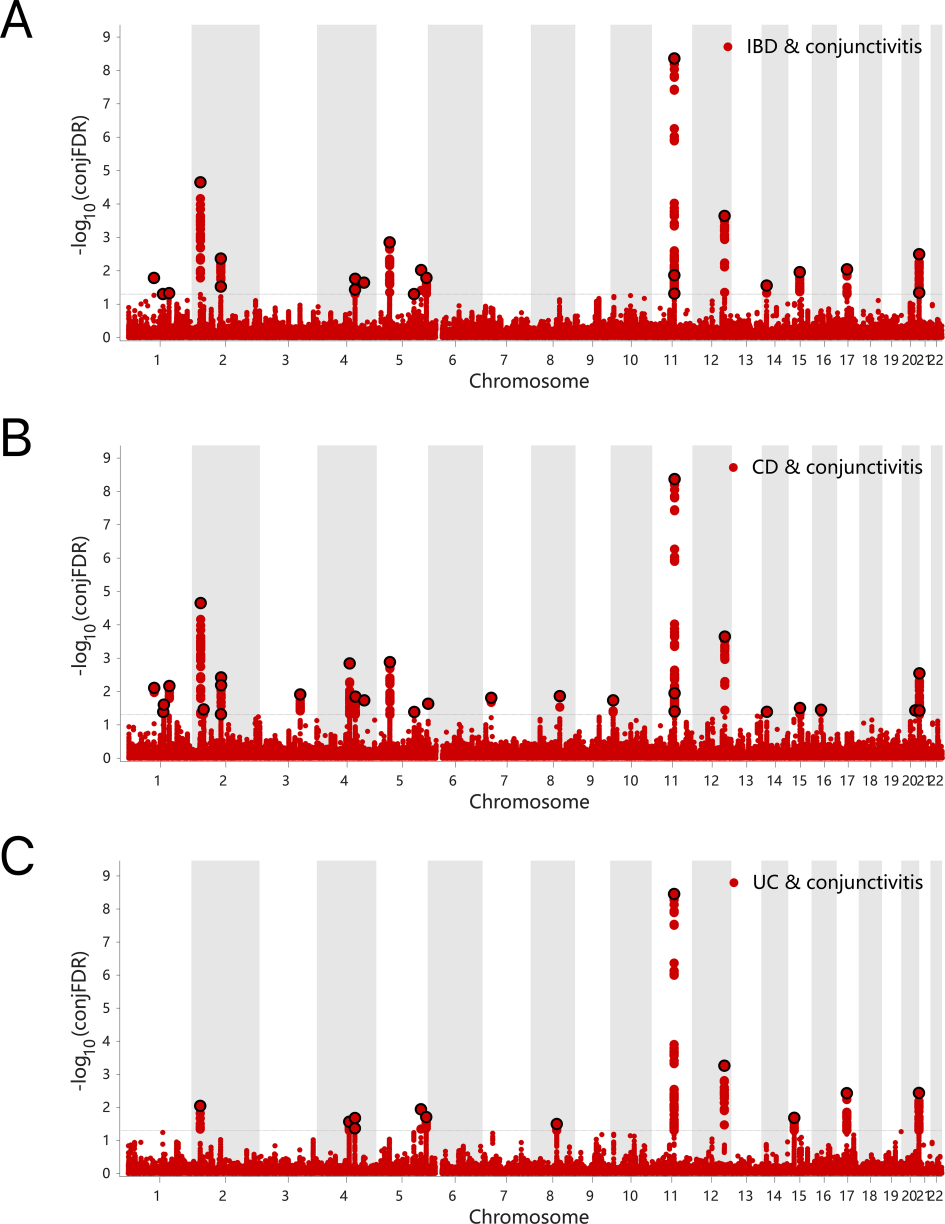
**(A)** ConjFDR Manhattan plot of IBD and conjunctivitis. **(B)** ConjFDR Manhattan plot of CD and conjunctivitis. **(B)** ConjFDR Manhattan plot of UC and conjunctivitis. The shared risk loci between conjunctivitis and IBD, CD and UC were marked. The statistically significant causality is defined to be conjFDR <0.05. IBD, inflammatory bowel disease; CD, Crohn’s disease; UC, ulcerative colitis.

The results of the ConjFDR analysis identified common loci between the two traits with a high level of confidence. Specifically, 17 common genetic risk loci were identified for IBD and conjunctivitis according to the screening criterion “ConjFDR < 0.05”, of which 14 acted in the same direction for both diseases, while the remaining 3 acted in the opposite direction (**Figure 5A**, **Supplementary Table S10**). Similarly, 24 common risk loci were identified for CD and conjunctivitis, with 19 acting in the same direction (**Figure 5B**, **Supplementary Table S11**). Besides, UC and conjunctivitis shared the fewest number of genetic risk loci (11) with 7 acting in the same direction (**Figure 5C**, **Supplementary Table S12**).

### 3.4 Functional annotations

**Figure 6:**
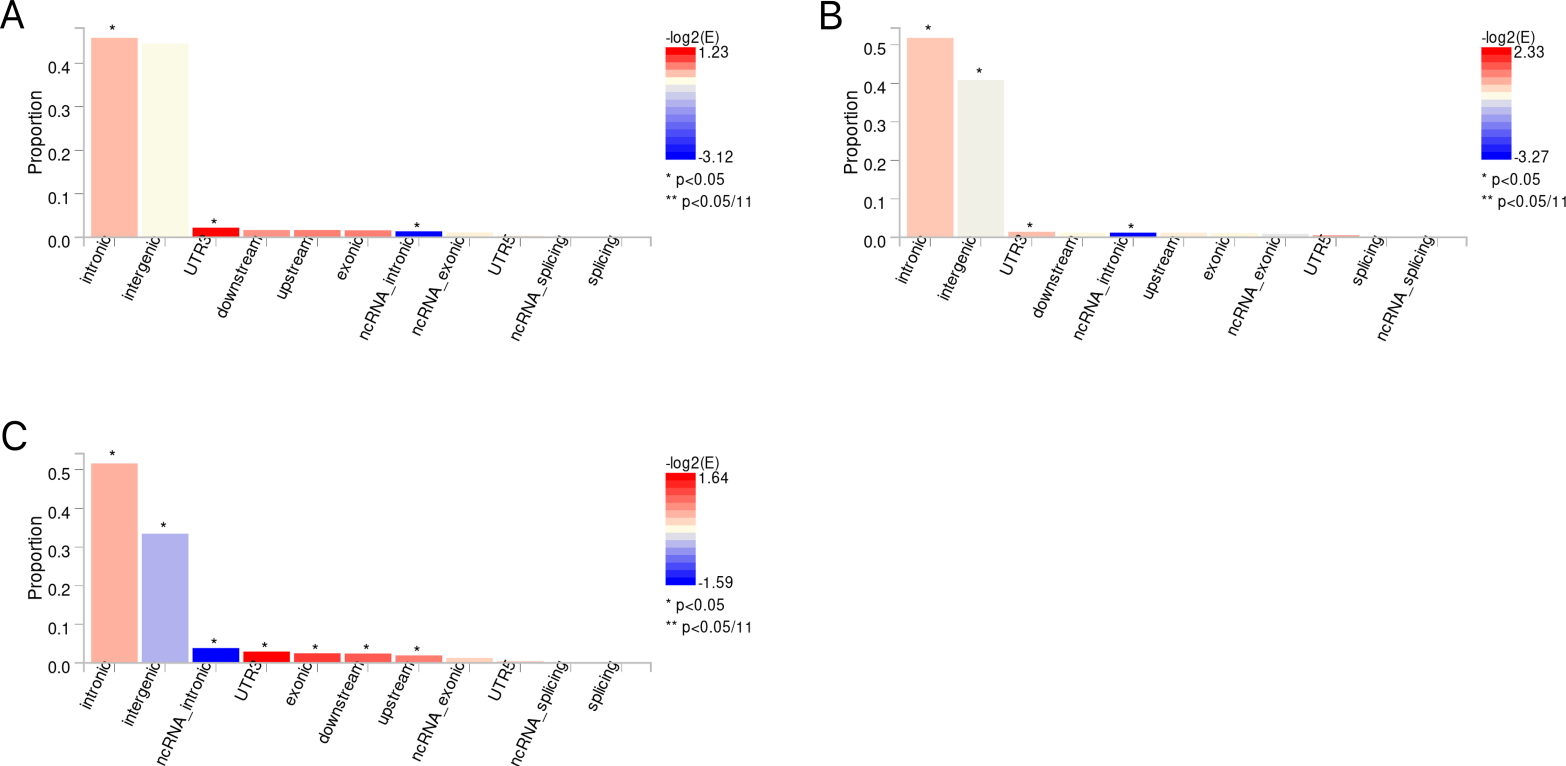
**(A)** The distribution of functional attributes of candidate SNPS between IBD and conjunctivitis. **(B)** The distribution of functional attributes of candidate SNPS between CD and conjunctivitis. **(C)** The distribution of functional attributes of candidate SNPS between UC and conjunctivitis. IBD, inflammatory bowel disease; CD, Crohn’s disease; UC, ulcerative colitis.

**Figure 7:**
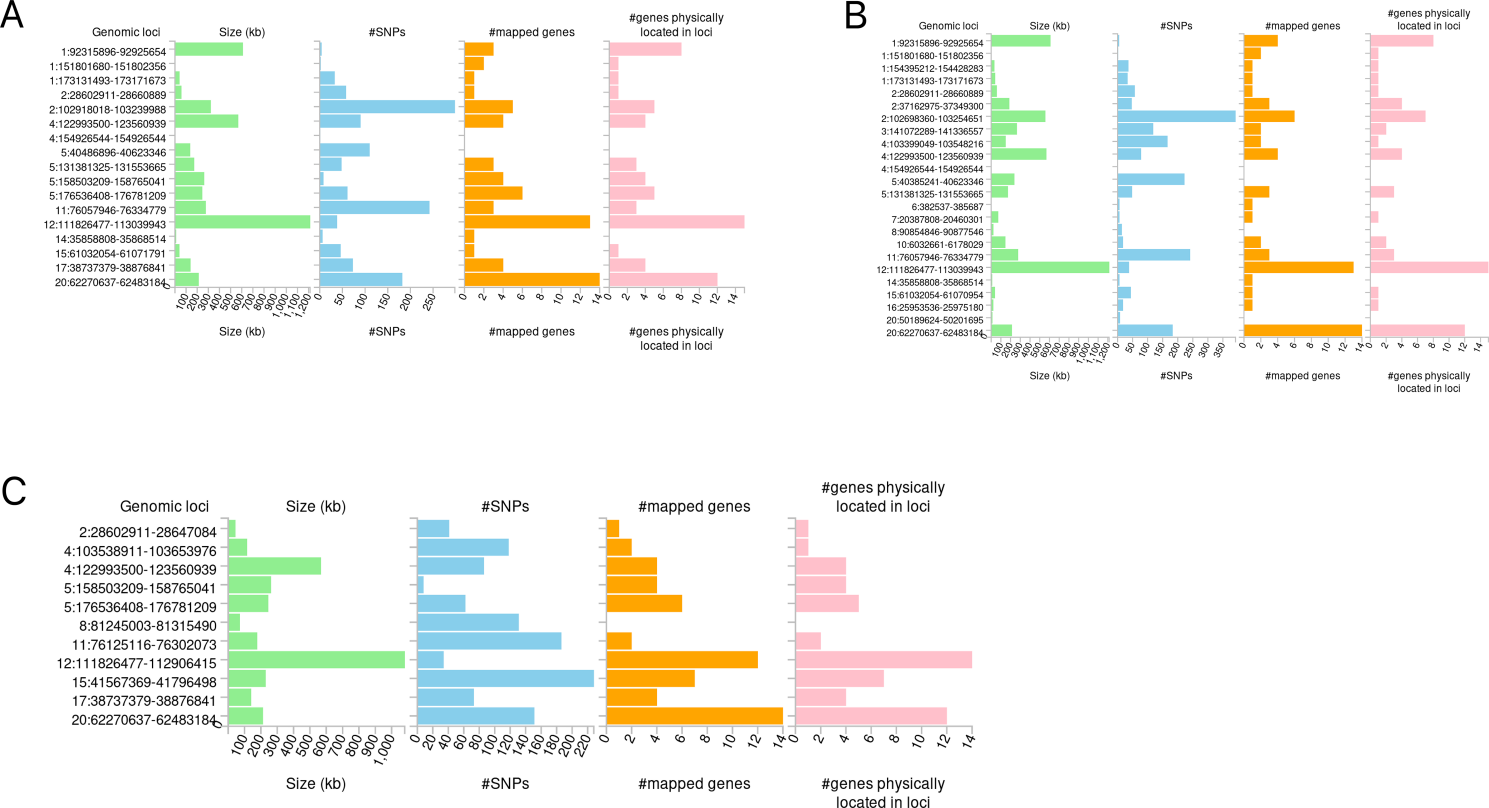
Distribution of mapping genes on chromosomes. **(A)** IBD-conjunctivitis. **(B)** CD-conjunctivitis. **(C)** UC-conjunctivitis.

The candidate SNPs shared by IBD (including CD and UC) with conjunctivitis were 1300, 1787, and 1118, respectively, with predominantly intronic and intergenic functional attributes (**Figures 6A–C**, **Supplementary Tables S13–S15**). In addition, mapping genes were observed between them. Specifically, 65, 66, and 56 mapping genes were enriched for IBD, CD, and UC with conjunctivitis, respectively (**Supplementary Tables S16–18**), which were more concentrated and distributed on chromosome 12 (**Figures 7A–C**).

**Figure 8:**
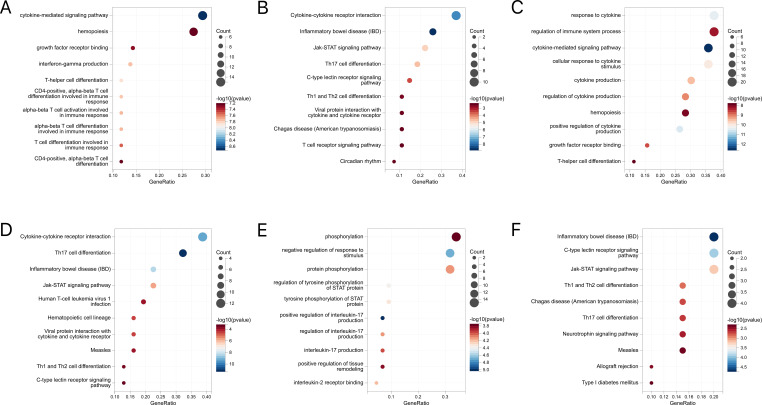
**(A)** GO enrichment analysis of mapped genes between IBD and conjunctivitis. **(B)** KEGG enrichment analysis of mapped genes between IBD and conjunctivitis. **(C)** GO enrichment analysis of mapped genes between CD and conjunctivitis. **(D)** KEGG enrichment analysis of mapped genes between CD and conjunctivitis. **(E)** GO enrichment analysis of mapped genes between UC and conjunctivitis. **(F)** KEGG enrichment analysis of mapped genes between UC and conjunctivitis. IBD, inflammatory bowel disease; CD, Crohn’s disease; UC, ulcerative colitis.

Further, these mapping genes were subjected to the GO and KEGG pathway enrichment analyses (**Figures 8A-F**). The GO enrichment analysis suggested their correlation with cytokine-mediated and immune responses. While IBD and CD were associated with helper T-cell differentiation, UC showed tyrosine phosphorylation of STAT proteins and the regulation of the interleukin-17 and interleukin-2 receptors. The KEGG analysis generated similar results, where the JAK-STAT signaling pathway, Th17 cell differentiation, and Th1 and Th2 cell differentiation pathways were highlighted.

### 3.5 MTAG

**Figure 9:**
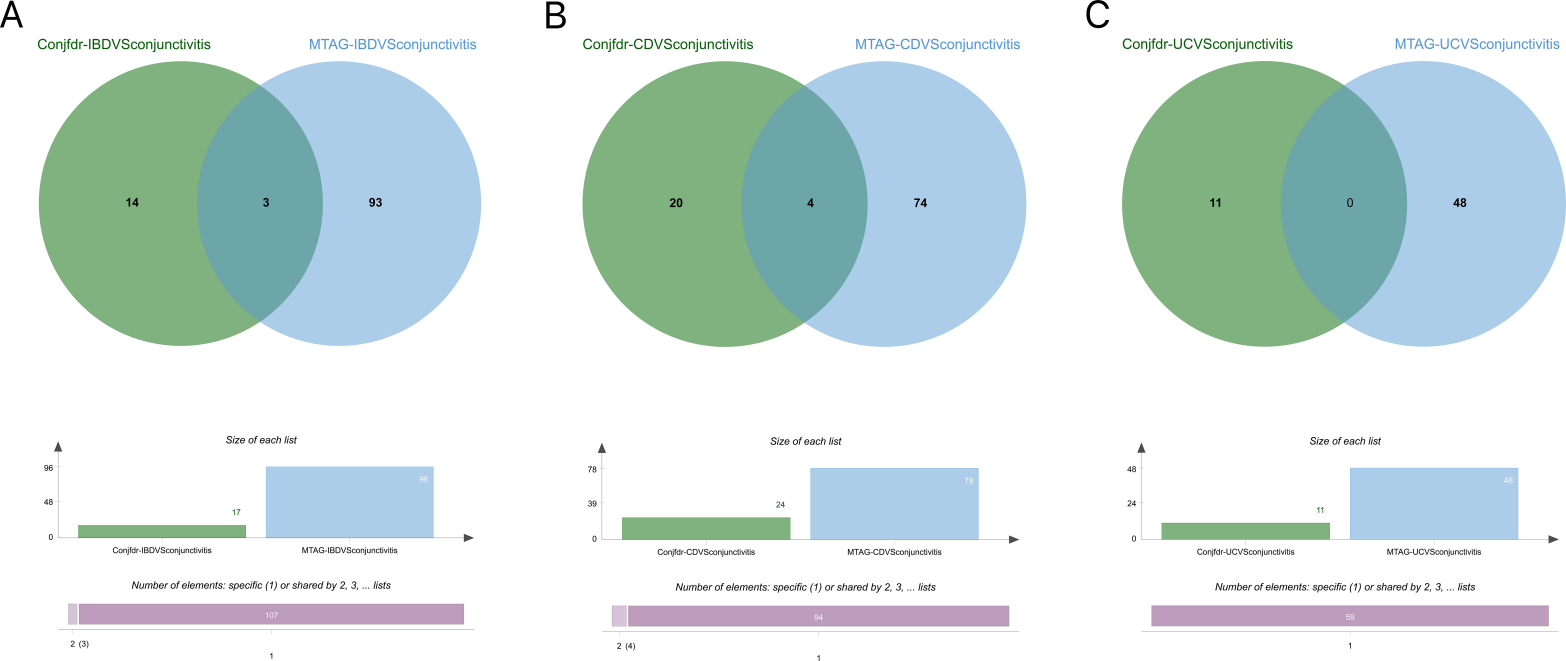
**(A)** Intersection gene map of IBD and conjunctivitis after conjfdr and MTAG analysis. **(B)** Intersection gene map of CD and conjunctivitis after conjfdr and MTAG analysis. **(C)** Intersection gene map of UC and conjunctivitis after conjfdr and MTAG analysis. IBD, inflammatory bowel disease; CD, Crohn’s disease; UC, ulcerative colitis.

Following MTAG analysis and Fuma annotation, a total of 96 genetic risk loci were identified between IBD and conjunctivitis (**Supplementary Table S19**). Validation results indicated that three genes (RORC, AC10469.5.3, and AC10810.1) were corroborated by both conjfdr and MTAG analyses (**Figure 9A**). Subsequent to MTAG analysis, 78 genetic risk loci were uncovered in CD (**Supplementary Table S20**), with validation of four genes (AC10469.5.3, ZBTB38, AC10810.1, and IL2RA) evident in the findings (**Figure 9B**). In the case of UC, a total of 48 genetic risk loci were obtained (**Supplementary Table S21**), with no shared genes identified through validation processes (**Figure 9C**).

## 4 Discussion

Our study results provided an overall assessment of the epidemiology of IBD (including CD and UC) and conjunctivitis and identified a similar genetic structure between them. Results revealed that IBD (including CD and UC) and conjunctivitis were genetically correlated overall while showing localized correlation on chromosome 11. Moreover, IBD (including CD and UC) had positive causality for conjunctivitis; however, the reverse phenomenon was not observed. In addition, condFDR/conjFDR analyses identified 17 genetic risk loci between IBD and conjunctivitis, whereas 24 and 11 genetic risk loci were shared between CD and UC with conjunctivitis, respectively. Finally, their mapping genes were associated with immune and cytokine regulation. Collectively, these results suggested that the genetic association between IBD (including CD and UC) and conjunctivitis was mainly due to overlapping genetic structures and causal effects.

The association between IBD and conjunctivitis is well established. According to a prospective study involving 116 patients with IBD, 34 developed ocular abnormalities, and 10 were affected with conjunctivitis (36). Another study also reported that IBD could lead to conjunctivitis (37), and meta-analyses on the relationship between IBD and conjunctivitis also reported consistent results (38). However, contradictory findings were also reported. A study involving a French population of IBD patients revealed that ocular inflammatory conditions, such as conjunctivitis were not associated with IBD (39). Nevertheless, several of the above-mentioned studies were influenced by confounding factors, such as the use of immunomodulators and/or anti-TNF drugs. The present study investigated a genetic perspective involving the SNPs, thereby effectively avoiding the confounding factors. Moreover, our findings highlighted the causal association of the two diseases and provided evidence of their genetic structure for co-morbidities. Additionally, the genetic association of conjunctivitis was more pronounced with CD than with UC, as confirmed by previous reports (38, 40). While CD is characterized by transmural inflammation of the intestine that can affect any part of the gastrointestinal tract from the oral cavity to the perineum, UC is limited to the mucosal layer of the colon (2). In addition, the CD has important immunologic differences compared with UC (41). These differences between the two subtypes of IBD may contribute to the aforementioned discrepancies.

The concept of the “gut-retina” axis has been one of the current hotspots of biological research since its introduction (42). It is closely related to the homeostasis of the ocular immune system and is critically involved in various ocular diseases, such as conjunctivitis, uveitis, and diabetic retinopathy (43). The “gut-retina” axis has become a new area of basic and clinical research in ophthalmology. Some studies have also reported the possible pathways for the formation and alteration of intestinal flora-ocular surface-lacrimal gland axis (44). Since IBD and conjunctivitis are immune-mediated disorders and are inter-connected, they may form this “gut-retina” axis in which genetic structural overlap may be a potential basis for the mechanism of action involved.

Next, the enrichment analysis of IL17 and Th17 obtained significant results. IL-17, a well-known pro-inflammatory factor, plays an important role in response to injury, physiological stress, and infection, thus maintaining health (45). Recent epidemiological studies indicate that serum IL-17 levels are significantly higher in patients with UC, CD, and vernal keratoconjunctivitis compared to healthy individuals, suggesting IL-17’s potential as a biomarker for inflammatory diseases (46, 47). In addition, the IL-17 cytokine axis is associated with diseases affecting the eyes and the gut (48). Tool-targeted IL-17 pathways may be of great importance in patients with hormone-resistant conjunctivitis (49). In a recent retrospective study, IL-17 inhibitors cured 24 patients with new-onset IBD (50). Moreover, Th17, a T-cell lineage distinct from Th1 and Th2 cells, is a novel type of pre-inflammatory T effector cell (51). In a recent mouse model of allergic conjunctivitis, stimulation and activation of the Th17 cytokines IL-17A and IL-17F, as well as the specific transcription factor RORγt, suggest that developmental enhancement can exacerbate Th2 dominant allergic inflammation in conjunctivitis (52). In addition, inhibition of the Th17 differentiation relieved the inflammatory symptoms of IBD (53).

The genes identified through conjfdr and MTAG analyses are worthy of attention. RORC is a protein-coding gene that regulates the polarization and function of Th17 cells, and is associated with autoimmune diseases and inflammation (54). RORC can facilitate the production of IL21, IL22, and IL17, thereby stimulating Th17 cells to maintain the inflammatory response in IBD (55). In a murine model, it was discovered that the methylation of RORC regulates the changes in Th1/Th17 cells to participate in the immune response of conjunctivitis (56). As an inflammation-related lncRNA, AC104695.3 may be involved in the pathogenesis of inflammatory diseases such as IBD and conjunctivitis (57). In a recent GWAS study, ZBTB38 has been identified as a susceptibility gene for CD (58). In the genetic analysis of myopia control, it was discovered that ZBTB38 has an impact on the conjunctival area (59). IL2RA is an important regulatory factor of immune function. On one hand, IL2RA can increase the expression of CD25 and activate the IL-2 pathway in peripheral CD4 T cells. On the other hand, it can also enhance the responsiveness of CD4 T cells to T cell receptor stimulation, ultimately inducing IBD (60). The IL2RA enhancer variant can modulate the response to IL-2 signaling, thereby impacting the immune defense process of CD cells (61). The expression of IL2RA is closely associated with the severity of inflammation in the conjunctivitis mouse model (62).

Despite several significant findings, the present study had some limitations. First, it is impossible to completely negate the occurrence of LD. Although these methods (LDSC, HESS, MR, conjFDR,and MTAG) substantially reduced the possibility of sample overlap, the exaggeration of cross-trait enrichment results due to overlapping participants cannot be ruled out. In addition, certain unavoidable factors, such as behavioral, social, and environmental factors persisted. The current GWAS data involved individuals of European ancestry; therefore, the results cannot be generalized to the non-European populations. The statistical power of GWAS is contingent upon sample size. A larger sample size yields greater statistical power and identifies more loci of risk, thus justifying further scrutiny of larger independent cohorts in future studies.While experimental validation was not conducted, our findings can serve as a reference for future research on cell biology mechanisms.

## 5 Conclusion

In conclusion, this study expands the understanding of the genetic structure and causal relationship between IBD (including CD and UC) and conjunctivitis by contributing to the previous epidemiologic studies. Thus, our findings would benefit the current treatment of the comorbidity between IBD (including CD and UC) and conjunctivitis.

## Data Availability

The original contributions presented in the study are included in the article/**Supplementary Material**, further inquiries can be directed to the corresponding author.

## References

[1] Hodson R. Inflammatory bowel disease. Nature. (2016) 540:S97. doi: 10.1038/540S97a 28002398

[2] Flynn S Eisenstein S. Inflammatory bowel disease presentation and diagnosis. Surg Clin North Am. (2019) 99:1051–62. doi: 10.1016/j.suc.2019.08.001 31676047

[3] GBD 2017 Inflammatory Bowel Disease Collaborators. The global, regional, and national burden of inflammatory bowel disease in 195 countries and territories, 1990-2017: a systematic analysis for the Global Burden of Disease Study 2017. Lancet Gastroenterol Hepatol. (2020) 5:17–30. doi: 10.1016/S2468-1253(19)30333-4 31648971 PMC7026709

[4] Kilic Y Kamal S Jaffar F Sriranganathan D Quraishi MN Segal JP. Prevalence of extraintestinal manifestations in inflammatory bowel disease: A systematic review and meta-analysis. Inflammation Bowel Dis. (2024) 30:230–9. doi: 10.1093/ibd/izad061 37042969

[5] Azari AA Arabi A. Conjunctivitis: A systematic review. J Ophthal Vis Res. (2020) 15:372–95. doi: 10.18502/jovr.v15i3.7456 PMC7431717 32864068

[6] Ghanchi FD Rembacken BJ. Inflammatory bowel disease and the eye. Surv Ophthalmol. (2003) 48:663–76. doi: 10.1016/j.survophthal.2003.08.004 14609712

[7] Liu JZ van Sommeren S Huang H Ng SC Alberts R Takahashi A. Association analyses identify 38 susceptibility loci for inflammatory bowel disease and highlight shared genetic risk across populations. Nat Genet. (2015) 47:979–86. doi: 10.1038/ng.3359 PMC4881818 26192919

[8] Sakaue S Kanai M Tanigawa Y Karjalainen J Kurki M Koshiba S. A cross-population atlas of genetic associations for 220 human phenotypes. Nat Genet. (2021) 53:1415–24. doi: 10.1038/s41588-021-00931-x PMC12208603 34594039

[9] Li J Wang L Ma Y Liu Y. Inflammatory bowel disease and allergic diseases: A Mendelian randomization study. Pediatr Allergy Immunol. (2024) 35:e14147. doi: 10.1111/pai.14147 38773751

[10] Leonardi A Daull P Garrigue J-S Cavarzeran F Docquier M Di Stefano A. Conjunctival transcriptome analysis reveals the overexpression of multiple pattern recognition receptors in vernal keratoconjunctivitis. Ocul Surf. (2021) 19:241–8. doi: 10.1016/j.jtos.2020.09.009 33098984

[11] O’Connor LJ. The distribution of common-variant effect sizes. Nat Genet. (2021) 53:1243–9. doi: 10.1038/s41588-021-00901-3 34326547

[12] Andreassen OA Hindley GFL Frei O Smeland OB. New insights from the last decade of research in psychiatric genetics: discoveries, challenges and clinical implications. World Psychiatry. (2023) 22:4–24. doi: 10.1002/wps.21034 36640404 PMC9840515

[13] Zhang X Liang Y Huang Y Liu S Li Q Wang S. Evaluation of the observational associations and shared genetics between glaucoma with depression and anxiety. Invest Ophthalmol Vis Sci. (2024) 65:12. doi: 10.1167/iovs.65.3.12 PMC10929750 38466289

[14] Zhang J Qiu H Zhao Q Liao C Guoli Y Luo Q. Genetic overlap between schizophrenia and cognitive performance. Schizophr (Heidelb). (2024) 10:31. doi: 10.1038/s41537-024-00453-5 PMC10914834 38443399

[15] Guo J Luo Q Li C Liang H Cao Q Li Z. Evidence for the gut-skin axis: Common genetic structures in inflammatory bowel disease and psoriasis. Skin Res Technol. (2024) 30:e13611. doi: 10.1111/srt.13611 38348734 PMC10862160

[16] Bulik-Sullivan B Finucane HK Anttila V Gusev A Day FR Loh P-R. Psychiatric Genomics Consortium, Genetic Consortium for Anorexia Nervosa of the Wellcome Trust Case Control Consortium 3, Duncan L, et al. An atlas of genetic correlations across human diseases and traits. Nat Genet. (2015) 47:1236–41. doi: 10.1038/ng.3406 PMC4797329 26414676

[17] Shi H Mancuso N Spendlove S Pasaniuc B. Local genetic correlation gives insights into the shared genetic architecture of complex traits. Am J Hum Genet. (2017) 101:737–51. doi: 10.1016/j.ajhg.2017.09.022 PMC5673668 29100087

[18] Davey Smith G Hemani G. Mendelian randomization: genetic anchors for causal inference in epidemiological studies. Hum Mol Genet. (2014) 23:R89–98. doi: 10.1093/hmg/ddu328 PMC4170722 25064373

[19] Smeland OB Frei O Shadrin A O’Connell K Fan C-C Bahrami S. Discovery of shared genomic loci using the conditional false discovery rate approach. Hum Genet. (2020) 139:85–94. doi: 10.1007/s00439-019-02060-2 31520123

[20] Turley P Walters RK Maghzian O Okbay A Lee JJ Fontana MA. Multi-trait analysis of genome-wide association summary statistics using MTAG. Nat Genet. (2018) 50:229–37. doi: 10.1038/s41588-017-0009-4 PMC5805593 29292387

[21] Kurki MI Karjalainen J Palta P Sipilä TP Kristiansson K Donner KM. FinnGen provides genetic insights from a well-phenotyped isolated population. Nature. (2023) 613:508–18. doi: 10.1038/s41586-022-05473-8 PMC9849126 36653562

[22] Bulik-Sullivan BK Loh P-R Finucane HK Ripke S Yang J. Schizophrenia Working Group of the Psychiatric Genomics Consortium, Patterson N, Daly MJ, Price AL, Neale BM. LD Score regression distinguishes confounding from polygenicity in genome-wide association studies. Nat Genet. (2015) 47:291–5. doi: 10.1038/ng.3211 PMC4495769 25642630

[23] 1000 Genomes Project Consortium Auton A Brooks LD Durbin RM Garrison EP Kang HM. A global reference for human genetic variation. Nature. (2015) 526:68–74. doi: 10.1038/nature15393 26432245 PMC4750478

[24] Emdin CA Khera AV Kathiresan S. Mendelian randomization. JAMA. (2017) 318:1925–6. doi: 10.1001/jama.2017.17219 29164242

[25] Slob EAW Burgess S. A comparison of robust Mendelian randomization methods using summary data. Genet Epidemiol. (2020) 44:313–29. doi: 10.1002/gepi.22295 PMC7317850 32249995

[26] Burgess S Thompson SG. Interpreting findings from Mendelian randomization using the MR-Egger method. Eur J Epidemiol. (2017) 32:377–89. doi: 10.1007/s10654-017-0255-x PMC5506233 28527048

[27] Bowden J Davey Smith G Haycock PC Burgess S. Consistent estimation in mendelian randomization with some invalid instruments using a weighted median estimator. Genet Epidemiol. (2016) 40:304–14. doi: 10.1002/gepi.21965 PMC4849733 27061298

[28] Milligan BG. Maximum-likelihood estimation of relatedness. Genetics. (2003) 163:1153–67. doi: 10.1093/genetics/163.3.1153 PMC1462494 12663552

[29] Verbanck M Chen C-Y Neale B Do R. Detection of widespread horizontal pleiotropy in causal relationships inferred from Mendelian randomization between complex traits and diseases. Nat Genet. (2018) 50:693–8. doi: 10.1038/s41588-018-0099-7 PMC6083837 29686387

[30] Luo Q Zhou P Chang S Huang Z Zhu Y. The gut-lung axis: Mendelian randomization identifies a causal association between inflammatory bowel disease and interstitial lung disease. Heart Lung. (2023) 61:120–6. doi: 10.1016/j.hrtlng.2023.05.016 37247539

[31] Burgess S Bowden J Fall T Ingelsson E Thompson SG. Sensitivity analyses for robust causal inference from mendelian randomization analyses with multiple genetic variants. Epidemiology. (2017) 28:30–42. doi: 10.1097/EDE.0000000000000559 27749700 PMC5133381

[32] Frei O Holland D Smeland OB Shadrin AA Fan CC Maeland S. Bivariate causal mixture model quantifies polygenic overlap between complex traits beyond genetic correlation. Nat Commun. (2019) 10:2417. doi: 10.1038/s41467-019-10310-0 31160569 PMC6547727

[33] Liley J Wallace C. A pleiotropy-informed Bayesian false discovery rate adapted to a shared control design finds new disease associations from GWAS summary statistics. PloS Genet. (2015) 11:e1004926. doi: 10.1371/journal.pgen.1004926 25658688 PMC4450050

[34] Andreassen OA Djurovic S Thompson WK Schork AJ Kendler KS O’Donovan MC. Improved detection of common variants associated with schizophrenia by leveraging pleiotropy with cardiovascular-disease risk factors. Am J Hum Genet. (2013) 92:197–209. doi: 10.1016/j.ajhg.2013.01.001 23375658 PMC3567279

[35] Shen W Song Z Zhong X Huang M Shen D Gao P. Sangerbox: A comprehensive, interaction-friendly clinical bioinformatics analysis platform. iMeta. (2022) 1:e36. doi: 10.1002/imt2.36 38868713 PMC10989974

[36] Yilmaz S Aydemir E Maden A Unsal B. The prevalence of ocular involvement in patients with inflammatory bowel disease. Int J Colorectal Dis. (2007) 22:1027–30. doi: 10.1007/s00384-007-0275-1 17262200

[37] Felekis T Katsanos K Kitsanou M Trakos N Theopistos V Christodoulou D. Spectrum and frequency of ophthalmologic manifestations in patients with inflammatory bowel disease: a prospective single-center study. Inflammation Bowel Dis. (2009) 15:29–34. doi: 10.1002/ibd.20584 18626979

[38] Li J-X Chiang C-C Chen S-N Lin J-M Tsai Y-Y. The prevalence of ocular extra-intestinal manifestations in adults inflammatory bowel disease: A systematic review and meta-analysis. Int J Environ Res Public Health. (2022) 19:15683. doi: 10.3390/ijerph192315683 36497759 PMC9737331

[39] Cloché V Buisson A Tréchot F Batta B Locatelli A Favel C. Ocular symptoms are not predictive of ophthalmologic inflammation in inflammatory bowel disease. Digestl Liver Dis. (2013) 45:195–9. doi: 10.1016/j.dld.2012.10.013 23200464

[40] Salmon JF Wright JP Murray ADN. Ocular inflammation in crohn’s disease. Ophthalmology. (1991) 98:480–4. doi: 10.1016/S0161-6420(91)32268-1 2052301

[41] Vavricka SR Brun L Ballabeni P Pittet V Prinz Vavricka BM Zeitz J. Frequency and risk factors for extraintestinal manifestations in the Swiss inflammatory bowel disease cohort. Am J Gastroenterol. (2011) 106:110–9. doi: 10.1038/ajg.2010.343 20808297

[42] Qin X Zou H Niu C. The STING pathway: An uncharacterized angle beneath the gut-retina axis. Exp Eye Res. (2022) 217:108970. doi: 10.1016/j.exer.2022.108970 35114214

[43] Rowan S Jiang S Korem T Szymanski J Chang M-L Szelog J. Involvement of a gut-retina axis in protection against dietary glycemia-induced age-related macular degeneration. Proc Natl Acad Sci U.S.A. (2017) 114:E4472–81. doi: 10.1073/pnas.1702302114 PMC5465926 28507131

[44] Moon J Yoon CH Choi SH Kim MK. Can gut microbiota affect dry eye syndrome? Int J Mol Sci. (2020) 21:8443. doi: 10.3390/ijms21228443 33182758 PMC7697210

[45] McGeachy MJ Cua DJ Gaffen SL. The IL-17 family of cytokines in health and disease. Immunity. (2019) 50:892–906. doi: 10.1016/j.immuni.2019.03.021 30995505 PMC6474359

[46] Menesy A Hammad M Aref S Abozeid FAM. Level of interleukin 17 in inflammatory bowel disease and its relation with disease activity. BMC Gastroenterol. (2024) 24:135. doi: 10.1186/s12876-024-03218-7 38622545 PMC11020998

[47] Zicari AM Nebbioso M Zicari A Mari E Celani C Occasi F. Serum levels of IL-17 in patients with vernal keratoconjunctivitis: a preliminary report. Eur Rev Med Pharmacol Sci. (2013) 17:1242–4. 23690194

[48] Bridgewood C Newton D Bragazzi N Wittmann M McGonagle D. Unexpected connections of the IL-23/IL-17 and IL-4/IL-13 cytokine axes in inflammatory arthritis and enthesitis. Semin Immunol. (2021) 58:101520. doi: 10.1016/j.smim.2021.101520 34799224

[49] Meng X-T Shi Y-Y Zhang H Zhou H-Y. The role of th17 cells and IL-17 in th2 immune responses of allergic conjunctivitis. J Ophthalmol. (2020) 2020:e6917185. doi: 10.1155/2020/6917185 PMC7267877 32566265

[50] Letarouilly J-G Pham T Pierache A Acquacalda É Banneville B Barbarot S. New-onset inflammatory bowel diseases among IL-17 inhibitor-treated patients: results from the case-control MISSIL study. Rheumatol (Oxford). (2022) 61:2848–55. doi: 10.1093/rheumatology/keab819 34730790

[51] Vocca L Di Sano C Uasuf CG Sala A Riccobono L Gangemi S. IL-33/ST2 axis controls Th2/IL-31 and Th17 immune response in allergic airway diseases. Immunobiology. (2015) 220:954–63. doi: 10.1016/j.imbio.2015.02.005 25747940

[52] Wang Y-H Voo KS Liu B Chen C-Y Uygungil B Spoede W. A novel subset of CD4(+) T(H)2 memory/effector cells that produce inflammatory IL-17 cytokine and promote the exacerbation of chronic allergic asthma. J Exp Med. (2010) 207:2479–91. doi: 10.1084/jem.20101376 PMC2964570 20921287

[53] Gu Z Chen X Zhu D Wu S Yu C. Histone deacetylase 1 and 3 inhibitors alleviate colon inflammation by inhibiting Th17 cell differentiation. J Clin Lab Anal. (2022) 36:e24699. doi: 10.1002/jcla.24699 36106389 PMC9550981

[54] Yahia-Cherbal H Rybczynska M Lovecchio D Stephen T Lescale C Placek K. NFAT primes the human RORC locus for RORγt expression in CD4+ T cells. Nat Commun. (2019) 10:4698. doi: 10.1038/s41467-019-12680-x 31619674 PMC6795897

[55] Fransen K van Sommeren S Westra H-J Veenstra M Lamberts LE Modderman R. Correlation of genetic risk and messenger RNA expression in a th17/IL23 pathway analysis in inflammatory bowel disease. Inflamml Bowel Dis. (2014) 20:777–82. doi: 10.1097/MIB.0000000000000013 24662057

[56] Qiu Y Zhu Y Yu H Zhou C Kijlstra A Yang P. Dynamic DNA methylation changes of tbx21 and rorc during experimental autoimmune uveitis in mice. Mediators Inflammation. (2018) 2018:9129163. doi: 10.1155/2018/9129163 PMC6142759 30254507

[57] Zhang S Li X Tang C Kuang W. Inflammation-related long non-coding RNA signature predicts the prognosis of gastric carcinoma. Front Genet. (2021) 12:736766. doi: 10.3389/fgene.2021.736766 34819945 PMC8607501

[58] Kim K Oh SJ Lee J Kwon A Yu C-Y Kim S. Regulatory variants on the leukocyte immunoglobulin-like receptor gene cluster are associated with Crohn’s disease and interact with regulatory variants for TAP2. J Crohns Colitis. (2024) 18:47–53. doi: 10.1093/ecco-jcc/jjad127 37523193

[59] Alvarez-Peregrina C Sánchez-Tena MÁ Martinez-Perez C Santiago-Dorrego C Yvert T Andreu-Vazquez C. The influence of genetics in myopia control: A pilot study. J Clin Med. (2021) 10:808. doi: 10.3390/jcm10040808 33671267 PMC7922351

[60] Joosse ME Charbit-Henrion F Boisgard R Raatgeep R Lindenbergh-Kortleve DJ Costes LMM. Duplication of the IL2RA locus causes excessive IL-2 signaling and may predispose to very early onset colitis. Mucosal Immunol. (2021) 14:1172–82. doi: 10.1038/s41385-021-00423-5 PMC8379074 34226674

[61] Goldberg R Clough JN Roberts LB Sanchez J Kordasti S Petrov N. A Crohn’s disease-associated IL2RA enhancer variant determines the balance of T cell immunity by regulating responsiveness to IL-2 signalling. J Crohns Colitis. (2021) 15:2054–65. doi: 10.1093/ecco-jcc/jjab103 PMC8684452 34120187

[62] De Paiva CS Hwang CS Pitcher JD Pangelinan SB Rahimy E Chen W. Age-related T-cell cytokine profile parallels corneal disease severity in Sjogren’s syndrome-like keratoconjunctivitis sicca in CD25KO mice. Rheumatol (Oxford). (2010) 49:246–58. doi: 10.1093/rheumatology/kep357 PMC2909796 20007286

