# Supplementary material for: Genetic association and causal effects between inflammatory bowel disease and conjunctivitis
Source: Front Immunol. 2024 Sep 4;15:1409146. doi: 10.3389/fimmu.2024.1409146 (PMC11408187; doi:10.3389/fimmu.2024.1409146)
Supplement: Supplementary file 2 [file DataSheet2.docx]

**STROBE-MR checklist of recommended items to address in reports of Mendelian randomization studies**^1^ ^2^

| **Item No.** | **Section** | **Checklist item** | **Page No.** | **Relevant text from manuscript** |
| --- | --- | --- | --- | --- |
| 1 | **TITLE and ABSTRACT** | Indicate Mendelian randomization (MR) as the study’s design in the title and/or the abstract if that is a main purpose of the study |  | The causal effect analysis was performed using four methods of Mendelian randomization (MR) |
|  | **INTRODUCTION** |  |  |  |
| 2 | **Background** | Explain the scientific background and rationale for the reported study. What is the exposure? Is a potential causal relationship between exposure and outcome plausible? Justify why MR is a helpful method to address the study question |  | Presently, the co-morbidity of IBD and conjunctivitis is one of the major public health concerns. Therefore, analysis of the genetic perspective of their common genetic risk loci may be effective in disease management. |
| 3 | **Objectives** | State specific objectives clearly, including pre-specified causal hypotheses (if any). State that MR is a method that, under specific assumptions, intends to estimate causal effects |  | Since Mendelian randomization (MR), also known as a natural randomized controlled trial (RCT), is based on the "random assignment of parental alleles to offspring", it eliminates the possible confounding factors. Therefore, MR was used for causality analysis in the present study |
|  | **METHODS** |  |  |  |
| 4 | **Study design and data sources** | Present key elements of the study design early in the article. Consider including a table listing sources of data for all phases of the study. For each data source contributing to the analysis, describe the following: |  |  |
|  | a) | Setting: Describe the study design and the underlying population, if possible. Describe the setting, locations, and relevant dates, including periods of recruitment, exposure, follow-up, and data collection, when available. |  | NA |
|  | b) | Participants: Give the eligibility criteria, and the sources and methods of selection of participants. Report the sample size, and whether any power or sample size calculations were carried out prior to the main analysis |  | Three datasets, including IBD (ID: ebi-a-GCST004131, *N*_case_=25,042, *N*_control_=34,915), CD (ID: ebi-a-GCST004132, *N*_case_=12,194, *N*_control_=28,072), and UC (ID: ebi-a-GCST004133, *N*_case_=12,366, *N*_control_=33,609) from the IEU GWAS database (https://gwas.mrcieu.ac.uk/) were selected based on the sample size, number of SNPs, study ethnicity (Europe), and year of publication. Moreover, GWAS data for conjunctivitis (*N*_case_=32,417, *N*_control_=28,895) were obtained from the FinnGen database (https://r10.finngen.fi/) |
|  | c) | Describe measurement, quality control and selection of genetic variants |  | 1. In addition, pleiotropic (23,26), heterogeneity (27), and leave-one-out (28) analyses were performed to ensure the quality, accuracy, and reliability of the analyzed results |
|  | d) | For each exposure, outcome, and other relevant variables, describe methods of assessment and diagnostic criteria for diseases |  | NA |
|  | e) | Provide details of ethics committee approval and participant informed consent, if relevant |  | Not involved |
| 5 | **Assumptions** | Explicitly state the three core IV assumptions for the main analysis (relevance, independence and exclusion restriction) as well assumptions for any additional or sensitivity analysis |  | 1. Potential causal associations between IBD and conjunctivitis were evaluated using a bidirectional two-sample MR analysis following its three main assumptions. 2. In addition, pleiotropic (23,26), heterogeneity (27), and leave-one-out (28) analyses were performed to ensure the quality, accuracy, and reliability of the analyzed results. |
| 6 | **Statistical methods: main analysis** | Describe statistical methods and statistics used |  |  |
|  | a) | Describe how quantitative variables were handled in the analyses (i.e., scale, units, model) |  | NA |
|  | b) | Describe how genetic variants were handled in the analyses and, if applicable, how their weights were selected |  | NA |
|  | c) | Describe the MR estimator (e.g. two-stage least squares, Wald ratio) and related statistics. Detail the included covariates and, in case of two-sample MR, whether the same covariate set was used for adjustment in the two samples |  | In addition, the causal effects were estimated mainly by the inverse variance weighted (IVW) (22), while MR-Egger (23), weighted median (WM) (24), and maximum likelihood (ML) (25) methods supplemented the findings. |
|  | d) | Explain how missing data were addressed |  |  |
|  | e) | If applicable, indicate how multiple testing was addressed |  |  |
| 7 | **Assessment of assumptions** | Describe any methods or prior knowledge used to assess the assumptions or justify their validity |  | 1. In addition, the causal effects were estimated mainly by the inverse variance weighted (IVW) (22), while MR-Egger (23), weighted median (WM) (24), and maximum likelihood (ML) (25) methods supplemented the findings. |
| 8 | **Sensitivity analyses and additional analyses** | Describe any sensitivity analyses or additional analyses performed (e.g. comparison of effect estimates from different approaches, independent replication, bias analytic techniques, validation of instruments, simulations) |  | In addition, pleiotropic (23,26), heterogeneity (27), and leave-one-out (28) analyses were performed to ensure the quality, accuracy, and reliability of the analyzed results. |
| 9 | **Software and pre-registration** |  |  |  |
|  | a) | Name statistical software and package(s), including version and settings used |  | 1. The entire MR analysis utilized the TwoSampleMR R (https://mrcieu.github.io/TwoSampleMR/) and the MR-PRESSO R software packages (https://github.com/rondolab/MR-). 2. All the GWAS data and statistical software used in this study were publicly available (which can be accessed through the following URLs), and all the generated results in this study were provided in the main text and supplemental data. |
|  | b) | State whether the study protocol and details were pre-registered (as well as when and where) |  | Not involved |
|  | **RESULTS** |  |  |  |
| 10 | **Descriptive data** |  |  |  |
|  | a) | Report the numbers of individuals at each stage of included studies and reasons for exclusion. Consider use of a flow diagram |  | NA |
|  | b) | Report summary statistics for phenotypic exposure(s), outcome(s), and other relevant variables (e.g. means, SDs, proportions) |  | Three datasets, including IBD (ID: ebi-a-GCST004131, *N*_case_=25,042, *N*_control_=34,915), CD (ID: ebi-a-GCST004132, *N*_case_=12,194, *N*_control_=28,072), and UC (ID: ebi-a-GCST004133, *N*_case_=12,366, *N*_control_=33,609) from the IEU GWAS database (https://gwas.mrcieu.ac.uk/) were selected based on the sample size, number of SNPs, study ethnicity (Europe), and year of publication. Moreover, GWAS data for conjunctivitis (*N*_case_=32,417, *N*_control_=28,895) were obtained from the FinnGen database (https://r10.finngen.fi/) |
|  | c) | If the data sources include meta-analyses of previous studies, provide the assessments of heterogeneity across these studies |  | Not involved |
|  | d) | For two-sample MR:  i.  Provide justification of the similarity of the genetic variant-exposure associations between the exposure and outcome samples  ii.  Provide information on the number of individuals who overlap between the exposure and outcome studies |  | NA |
| 11 | **Main results** |  |  |  |
|  | a) | Report the associations between genetic variant and exposure, and between genetic variant and outcome, preferably on an interpretable scale |  | Tables 1–2 demonstrate the final results of the correlation analysis between IBD (including CD and UC) and conjunctivitis using four methods. The forward MR analysis (IBD and subtypes as exposure and conjunctivitis as outcome) suggested a positive causality in all three cases (p < 0.05, Figures 2A–C, Table 1). Conversely, the backward MR analysis did not demonstrate a causal effect of conjunctivitis on IBD or its subtypes (Table 2) |
|  | b) | Report MR estimates of the relationship between exposure and outcome, and the measures of uncertainty from the MR analysis, on an interpretable scale, such as odds ratio or relative risk per SD difference |  | \| Exposures \| Outcomes \| nSNPs \| Method \| OR（95%CI） \| P \| Heterogeneity test \| \| \| Pleiotropy  test \| F \| \| --- \| --- \| --- \| --- \| --- \| --- \| --- \| --- \| --- \| --- \| --- \| \| Method \| Q \| P \| P intercept \| \| IBD \| Conjunctivitis \| 90 \| IVW (mre) \| 1.05 (1.03-1.08) \| 7.49E-06 \| MR Egger \| 171.73 \| 2.73E-07 \| 0.55 \| 29.86-500.60 \| \|  \|  \|  \| WM \| 1.06(1.02-1.09) \| 3.68E-04 \| IVW \| 172.41 \| 2.82E-07 \|  \|  \| \|  \|  \|  \| MR Egger \| 1.04(0.97-1.10) \| 0.25 \|  \|  \|  \|  \|  \| \|  \|  \|  \| ML \| 1.06(1.04-1.07) \| 3.7E-02 \|  \|  \|  \|  \|  \| \| CD \| Conjunctivitis \| 73 \| IVW (mre) \| 1.04(1.02-1.06) \| 5.37E-04 \| MR Egger \| 161.90 \| 4.70E-09 \| 0.80 \| 30.15-489.58 \| \|  \|  \|  \| WM \| 1.02(1.00-1.05) \| 0.06 \| IVW \| 162.04 \| 6.91E-09 \|  \|  \| \|  \|  \|  \| MR Egger \| 1.03(0.97-1.09) \| 0.29 \|  \|  \|  \|  \|  \| \|  \|  \|  \| ML \| 1.04(1.02-1.05) \| 2.23E-07 \|  \|  \|  \|  \|  \| \| UC \| Conjunctivitis \| 47 \| IVW (mre) \| 1.03(1.00-1.06) \| 0.03 \| MR Egger \| 97.43 \| 9.83E-06 \| 0.74 \| 30.47-186.78 \| \|  \|  \|  \| WM \| 1.04(1.00-1.07) \| 0.02 \| IVW \| 97.67 \| 1.38E-05 \|  \|  \| \|  \|  \|  \| MR Egger \| 1.02(0.92-1.11) \| 0.04 \|  \|  \|  \|  \|  \| \|  \|  \|  \| ML \| 1.03(1.01-1.05) \| 9.49E-03 \|  \|  \|  \|  \|  \|  \| Exposures \| Outcomes \| nSNPs \| Method \| OR（95%CI） \| P \| Heterogeneity test \| \| \| Pleiotropy  test \| F \| \| --- \| --- \| --- \| --- \| --- \| --- \| --- \| --- \| --- \| --- \| --- \| \| Method \| Q \| P \| P intercept \| \| Conjunctivitis \| IBD \| 6 \| IVW (fe) \| 1.07(0.87-1.31) \| 0.51 \| MR Egger \| 4.00 \| 0.41 \| 0.75 \| 31.23-50.61 \| \|  \|  \|  \| WM \| 1.17(0.92-1.49) \| 0.19 \| IVW \| 4.12 \| 0.53 \|  \|  \| \|  \|  \|  \| MR Egger \| 0.89(0.29-2.67) \| 0.84 \|  \|  \|  \|  \|  \| \|  \|  \|  \| ML \| 1.07(0.88-1.31) \| 0.51 \|  \|  \|  \|  \|  \| \| Conjunctivitis \| CD \| 5 \| IVW (fe) \| 1.31(1.00-1.72) \| 0.70 \| MR Egger \| 3.42 \| 0.33 \| 0.38 \| 32.25-50.61 \| \|  \|  \|  \| WM \| 1.37(0.97-1.92) \| 0.34 \| IVW \| 4.63 \| 0.33 \|  \|  \| \|  \|  \|  \| MR Egger \| 3.16(0.58-17.27) \| 0.35 \|  \|  \|  \|  \|  \| \|  \|  \|  \| ML \| 1.32(1.00-1.73) \| 0.70 \|  \|  \|  \|  \|  \| \| Conjunctivitis \| UC \| 6 \| IVW (fe) \| 1.01(0.78-1.31) \| 0.81 \| MR Egger \| 2.53 \| 0.64 \| 0.74 \| 31.23-50.61 \| \|  \|  \|  \| WM \| 1.06(0.78-1.44) \| 0.83 \| IVW \| 2.66 \| 0.75 \|  \|  \| \|  \|  \|  \| MR Egger \| 1.30(0.32-5.30) \| 0.78 \|  \|  \|  \|  \|  \| \|  \|  \|  \| ML \| 1.01(0.78-1.31) \| 0.69 \|  \|  \|  \|  \|  \| |
|  | c) | If relevant, consider translating estimates of relative risk into absolute risk for a meaningful time period |  | The forward MR analysis (IBD and subtypes as exposure and conjunctivitis as outcome) suggested a positive causality in all three cases (p < 0.05, Figures 2A–C, Table 1). |
|  | d) | Consider plots to visualize results (e.g. forest plot, scatterplot of associations between genetic variants and outcome versus between genetic variants and exposure) |  | Further, in the leave-one-out analysis (Figures 3A–F), SNPs showed a concentrated distribution without evidence of any abnormal SNPs |
| 12 | **Assessment of assumptions** |  |  |  |
|  | a) | Report the assessment of the validity of the assumptions |  | 1.Further, in the leave-one-out analysis (Figures 3A–F), SNPs showed a concentrated distribution without evidence of any abnormal SNPs.  2. In addition, no bias in the weak instrumental variables was observed (F > 10). Collectively, these findings validated the reliability of the results. The F-statistics corresponding to all the instrumental variables were > 10, indicating no bias in the weak instrumental variables. |
|  | b) | Report any additional statistics (e.g., assessments of heterogeneity across genetic variants, such as *I^2^*, Q statistic or E-value) |  | In addition, no bias in the weak instrumental variables was observed (F > 10). Collectively, these findings validated the reliability of the results. The F-statistics corresponding to all the instrumental variables were > 10, indicating no bias in the weak instrumental variables. |
| 13 | **Sensitivity analyses and additional analyses** |  |  |  |
|  | a) | Report any sensitivity analyses to assess the robustness of the main results to violations of the assumptions |  | Further, in the leave-one-out analysis (Figures 3A–F), SNPs showed a concentrated distribution without evidence of any abnormal SNPs. |
|  | b) | Report results from other sensitivity analyses or additional analyses |  | In addition, no bias in the weak instrumental variables was observed (F > 10). Collectively, these findings validated the reliability of the results. The F-statistics corresponding to all the instrumental variables were > 10, indicating no bias in the weak instrumental variables. |
|  | c) | Report any assessment of direction of causal relationship (e.g., bidirectional MR) |  | Tables 1–2 demonstrate the final results of the correlation analysis between IBD (including CD and UC) and conjunctivitis using four methods. The forward MR analysis (IBD and subtypes as exposure and conjunctivitis as outcome) suggested a positive causality in all three cases (p < 0.05, Figures 2A–C, Table 1). Conversely, the backward MR analysis did not demonstrate a causal effect of conjunctivitis on IBD or its subtypes (Table 2) |
|  | d) | When relevant, report and compare with estimates from non-MR analyses |  | The results of LDSC analysis of IBD and conjunctivitis suggested a genetic correlation Z-score of 4.58 and a rg value of 0.22 (p = 4.59e−06 < 0.05). Similarly, the Z-score and rg of CD were 4.28 and 0.22 (p = 1.84e−05 < 0.05), respectively. The rg for UC was smaller (0.16, p = 4.1e−03 < 0.05) than that of IBD and CD. Consequently, a positive association between either IBD or subtypes of IBD and conjunctivitis was observed. |
|  | e) | Consider additional plots to visualize results (e.g., leave-one-out analyses) |  | Further, in the leave-one-out analysis (Figures 3A–F), SNPs showed a concentrated distribution without evidence of any abnormal SNPs. The details of the instrumental variables used in this study are mentioned in Supplementary Tables S4–9. |
|  | **DISCUSSION** |  |  |  |
| 14 | **Key results** | Summarize key results with reference to study objectives |  | In conclusion, this study expands the understanding of the genetic structure and causal relationship between IBD (including CD and UC) and conjunctivitis by contributing to the previous epidemiologic studies. Thus, our findings would benefit the current treatment of the comorbidity between IBD (including CD and UC) and conjunctivitis. |
| 15 | **Limitations** | Discuss limitations of the study, taking into account the validity of the IV assumptions, other sources of potential bias, and imprecision. Discuss both direction and magnitude of any potential bias and any efforts to address them |  | Despite several significant findings, the present study had some limitations. First, it is impossible to completely negate the occurrence of LD. Although these methods (LDSC, HESS, MR, conjFDR,and MTAG) substantially reduced the possibility of sample overlap, the exaggeration of cross-trait enrichment results due to overlapping participants cannot be ruled out. In addition, certain unavoidable factors, such as behavioral, social, and environmental factors persisted. The current GWAS data involved individuals of European ancestry; therefore, the results cannot be generalized to the non-European populations.The statistical power of GWAS is contingent upon sample size. A larger sample size yields greater statistical power and identifies more loci of risk, thus justifying further scrutiny of larger independent cohorts in future studies.While experimental validation was not conducted, our findings can serve as a reference for future research on cell biology mechanisms. |
| 16 | **Interpretation** |  |  |  |
|  | a) | Meaning: Give a cautious overall interpretation of results in the context of their limitations and in comparison with other studies |  | The statistical power of GWAS is contingent upon sample size. A larger sample size yields greater statistical power and identifies more loci of risk, thus justifying further scrutiny of larger independent cohorts in future studies.While experimental validation was not conducted, our findings can serve as a reference for future research on cell biology mechanisms. |
|  | b) | Mechanism: Discuss underlying biological mechanisms that could drive a potential causal relationship between the investigated exposure and the outcome, and whether the gene-environment equivalence assumption is reasonable. Use causal language carefully, clarifying that IV estimates may provide causal effects only under certain assumptions |  | Next, the enrichment analysis of IL17 and Th17 obtained significant results. IL-17, a well-known pro-inflammatory factor, plays an important role in response to injury, physiological stress, and infection, thus maintaining health (43). Recent epidemiological studies indicate that serum IL-17 levels are significantly higher in patients with UC, CD, and vernal keratoconjunctivitis compared to healthy individuals, suggesting IL-17's potential as a biomarker for inflammatory diseases (44,45). In addition, the IL-17 cytokine axis is associated with diseases affecting the eyes and the gut (46). Tool-targeted IL-17 pathways may be of great importance in patients with hormone-resistant conjunctivitis (47). In a recent retrospective study, IL-17 inhibitors cured 24 patients with new-onset IBD (48). Moreover, Th17, a T-cell lineage distinct from Th1 and Th2 cells, is a novel type of pre-inflammatory T effector cell (49). In a recent mouse model of allergic conjunctivitis, stimulation and activation of the Th17 cytokines IL-17A and IL-17F, as well as the specific transcription factor RORγt, suggest that developmental enhancement can exacerbate Th2 dominant allergic inflammation in conjunctivitis (50). In addition, inhibition of the Th17 differentiation relieved the inflammatory symptoms of IBD (51). |
|  | c) | Clinical relevance: Discuss whether the results have clinical or public policy relevance, and to what extent they inform effect sizes of possible interventions |  | The association between IBD and conjunctivitis is well established. According to a prospective study involving 116 patients with IBD, 34 developed ocular abnormalities, and 10 were affected with conjunctivitis (33). Another study also reported that IBD could lead to conjunctivitis (34), and meta-analyses on the relationship between IBD and conjunctivitis also reported consistent results (35) |
| 17 | **Generalizability** | Discuss the generalizability of the study results (a) to other populations, (b) across other exposure periods/timings, and (c) across other levels of exposure |  | The current GWAS data involved individuals of European ancestry; therefore, the results cannot be generalized to the non-European populations. |
|  | **OTHER INFORMATION** |  |  |  |
| 18 | **Funding** | Describe sources of funding and the role of funders in the present study and, if applicable, sources of funding for the databases and original study or studies on which the present study is based |  | 11 Funding statement  This study did not receive any funding in any form. |
| 19 | **Data and data sharing** | Provide the data used to perform all analyses or report where and how the data can be accessed, and reference these sources in the article. Provide the statistical code needed to reproduce the results in the article, or report whether the code is publicly accessible and if so, where |  | All the GWAS data and statistical software used in this study were publicly available (which can be accessed through the following URLs), and all the generated results in this study were provided in the main text and supplemental data.  IEU database: https://gwas.mrcieu.ac.uk  FinnGen database (https://r10.finngen.fi/)  LDSC：https://github.com/bulik/ldsc  TwosampleMR: https://mrcieu.github.io/TwoSampleMR/  conjFDR: https://github.com/precimed/pleiofdr  FUMA: https://fuma.ctglab.nl  Sangerbox：http://vip.sangerbox.com/ |
| 20 | **Conflicts of Interest** | All authors should declare all potential conflicts of interest |  | The authors declare that the research was conducted in the absence of any commercial or financial relationships that could be construe as a potential conflict of interest. |

This checklist is copyrighted by the Equator Network under the Creative Commons Attribution 3.0 Unported (CC BY 3.0) license.

1. Skrivankova VW, Richmond RC, Woolf BAR, Yarmolinsky J, Davies NM, Swanson SA, et al. Strengthening the Reporting of Observational Studies in Epidemiology using Mendelian Randomization (STROBE-MR) Statement. JAMA. 2021;under review.

2. Skrivankova VW, Richmond RC, Woolf BAR, Davies NM, Swanson SA, VanderWeele TJ, et al. Strengthening the Reporting of Observational Studies in Epidemiology using Mendelian Randomisation (STROBE-MR): Explanation and Elaboration. BMJ. 2021;375:n2233.
